# Roux-en-Y Gastric Bypass Alters Brain Activity in Regions that Underlie Reward and Taste Perception

**DOI:** 10.1371/journal.pone.0125570

**Published:** 2015-06-03

**Authors:** Panayotis K. Thanos, Mike Michaelides, Mike Subrize, Mike L. Miller, Robert Bellezza, Robert N. Cooney, Lorenzo Leggio, Gene-Jack Wang, Ann M. Rogers, Nora D. Volkow, Andras Hajnal

**Affiliations:** 1 Behavioral Neuropharmacology and Neuroimaging Lab, Department of Psychology, Stony Brook University, Stony Brook, NY, United States of America; 2 Department of Neurosciences, Mt. Sinai Medical Center, NY, NY, United States of America; 3 Department. of Surgery, SUNY Upstate Medical University, Syracuse, NY, United States of America; 4 Section on Clinical Psychoneuroendocrinology and Neuropsychopharmacology, Laboratory of Clinical and Translational Studies, NIAAA, NIH, Bethesda, MD, United States of America; 5 Intramural Research Program, NIDA, NIH, Baltimore, MD, United States of America; 6 Center for Alcohol and Addiction Studies, Department of Behavioral and Social Sciences, Brown University, Providence, RI, United States of America; 7 NIAAA Intramural Research Program, NIH, Bethesda, MD, United States of America; 8 Department of Neural and Behavioral Sciences, Penn State University, Hershey, PA, United States of America; 9 Department of Surgery, Penn State University, Hershey, PA, United States of America; The University of Kansas Medical Center, UNITED STATES

## Abstract

**Background:**

Roux-en-Y gastric bypass (RYGB) surgery is a very effective bariatric procedure to achieve significant and sustained weight loss, yet little is known about the procedure’s impact on the brain. This study examined the effects of RYGB on the brain’s response to the anticipation of highly palatable versus regular food.

**Methods:**

High fat diet-induced obese rats underwent RYGB or sham operation and were then tested for conditioned place preference (CPP) for the bacon-paired chamber, relative to the chow-paired chamber. After CPP, animals were placed in either chamber without the food stimulus, and brain-glucose metabolism (BGluM) was measured using positron emission tomography (μPET).

**Results:**

Bacon CPP was only observed in RYGB rats that had stable weight loss following surgery. BGluM assessment revealed that RYGB selectively activated regions of the right and midline cerebellum (Lob 8) involved in subjective processes related to reward or expectation. Also, bacon anticipation led to significant activation in the medial parabrachial nuclei (important in gustatory processing) and dorsomedial tegmental area (key to reward, motivation, cognition and addiction) in RYGB rats; and activation in the retrosplenial cortex (default mode network), and the primary visual cortex in control rats.

**Conclusions:**

RYGB alters brain activity in areas involved in reward expectation and sensory (taste) processing when anticipating a palatable fatty food. Thus, RYGB may lead to changes in brain activity in regions that process reward and taste-related behaviors. Specific cerebellar regions with altered metabolism following RYGB may help identify novel therapeutic targets for treatment of obesity.

## Introduction

Obesity is a behavioral and metabolic disorder with devastating health consequences [1]. More than 64% of adult Americans are either overweight or obese [2,3].

Obesity is associated with impulsivity and impaired self-control for consumption of palatable foods. These behaviors are also implicated in the over-consumption of drugs and alcohol. The brain’s reward circuitry is involved in regulating normal and pathological eating and its dysfunction could contribute to obesity [4,5]. Thus, it is important to distinguish between hyperphagia induced by disruption in homeostatic mechanisms versus that induced by the hedonic properties of food and its activation of reward circuits.

The dopamine (DA) pathway plays an important role in reward, expectation of reward, and conditioning. Neurons originating in the ventral tegmental area (VTA) project to the nucleus accumbens (NAcc), prefrontal cortex (PFC), amygdala and hippocampus. In rodents, expectations for feeding and drinking are associated with accumbal DA increase [6,7]. In fasting humans, presentation of food-cues increased extracellular striatal DA, an effect associated with the subjective perception of desire for the food [8] and activated the VTA and striatum [9]. These observations suggest that food–cues stimulate the DA pathway, and its activation triggers motivation to consume food. Chemically stimulated food intake (norepinephrine microinjected into the hypothalamic paraventricular nucleus) in sated rats also increases DA release [10] and the taste of sucrose alone increases DA release in the NAcc of sham-fed rats [11]. The DA pathway has been implicated in obesity and eating disorders. For instance, blunted striatal activity is reported in obese subjects when presented with food-cues [12]. Decreases in striatal DA D2 receptors (D2R) have been reported in obese subjects [13], which are linked with decreased activity in ventral frontal regions [14]. Furthermore, body-mass-index (BMI) was negatively correlated with D2R in obese, but not lean subjects [13], and within 6 weeks following gastric bypass, D2R availability increased [15] though another study has reported decreases [16]. Similar negative correlation of D2R with obesity was reported in rats in which caloric restriction attenuated the D2R decline [17]. There is also evidence that obese subjects show enhanced regional brain activation to food-cues while showing blunted responses with food consumption [18]. These findings suggest that obesity is associated with changes in the reactivity of brain reward regions to food and food-cues [19,20]. The DA pathway also responds to conditioned-cues that predict rewarding stimuli; i.e. when an animal simply expects food [6,7]. Likewise, NAcc DA release increases when rats are presented with the cue alone [21] and in humans food cue exposure increases striatal DA [22,23] and activates the VTA and striatum [9]. Moreover, there is evidence that obese subjects show enhanced regional brain activation to food-cues while showing blunted responses with food consumption. Therefore, the neurobiology of cue-conditioning is essential to understand obesity and other eating disorders.

Here we examined the relationship between brain activity and food-reward anticipation, and how this relationship is impacted by Roux-en Y gastric bypass surgery (RYGB), following a chronic high-fat diet. Using a conditioned-place preference (CPP) procedure, we paired a familiar, high-fat food (bacon) with salient contextual cues (chamber attributes such as color and flooring). After the conditioning phase, animals were tested for CPP. Following this test, each animal’s brain-glucose metabolism (BGluM) was measured while exposed to the chamber without food (cues without stimulus). Scans were performed twice per animal, once in each chamber in counterbalanced order. Thereby, BGluM measurements captured each animal’s anticipation for food while exposed to each food-paired environment. Since bacon is a highly palatable food, a positive correlation between BGluM and preference for the bacon-paired chamber was expected, especially with regions associated with hunger and reward, notably the hypothalamus (homeostatic consumption) and NAcc (non-homeostatic consumption), respectively. We also hypothesized that RYGB influenced regional brain activation to food cues. Decreased food consumption following RYGB has been suggested to be in response to reduced reactivity to food cues [24–26]. However, reduced body adiposity and restricted meal-size following surgery would suggest instead greater reactivity as recently shown by our group in obese rats (fat diet induced) that underwent RYGB [27].

## Methods

### Animals

Fifty-eight Sprague-Dawley male rats (Taconic Farms, Inc., New York) were used. Twenty-six rats underwent RYGB surgery or Sham surgery (control group; n = 32) at 17 weeks of age. Eleven of the control rats were assigned to the *ad-libitum* high-fat (HF) group (Sham-AL), 12 were assigned to the pair-fed HF group (Sham-PF), and 9 were assigned to the *ad-libitum* normal diet group (Sham-ND). The *ad-libitum* group had unrestricted access to food and water. The pair-fed group had unlimited access to water, but their food was limited to the average intake of the RYGB group 24-hours earlier. At seven weeks of age, the animals’ body-weights were measured daily (1200-1400h), along with food and water intakes. The pair-fed group received 75% of their daily caloric intake (900-1100h) in the morning and the remaining 25% (1700-1900h) in the evening. The Sham-ND group received standard rat chow (13.5% kcal from fat; Purina Mills, Missouri). All other animals (RYGB, Sham-AL and Sham-PF groups) were given a HF-enriched diet starting at 6 weeks of age (60% kcal from fat) (Research Diets, Inc., New Jersey). Animals were originally on 12-hour light cycle (0800 hrs on, 2000 hrs off), then one week prior to the start of the experiment, animals were switched to a reverse 12-hour light cycle (2000 hrs on, 0800 hrs off). This was done so that all testing and handling were performed during the dark cycle when rodents are typically awake rather than the light cycle when they typically sleep. One week after light dark reversal was previously shown to be enough to restore baseline levels of locomotor activity as well as heart rate and blood pressure (107, 108). This study was carried out in strict accordance with the recommendations in the Guide for the Care and Use of Laboratory Animals of the National Institutes of Health. The protocol was approved by the Committee on the Ethics of Animal Experiments of Brookhaven National Lab (#: 212). All surgery was performed under ketamine xylazine anesthesia, and all efforts were made to minimize suffering.

### Surgery

All surgical procedures (sham or RYGB) were performed at Pennsylvania State University as previously described [[26]; S1 Materials]. Briefly, the sham surgery groups underwent anesthesia and laparotomy with intestinal manipulation but without forming anastomoses. Since diarrhea is a common side effect of gastric bypass in rats, and to reduce dehydration, experimental animals received subcutaneous lactated Ringer’s solution as needed. Postoperatively, Ceftriaxone (30 mg/kg SC) 2 times a day and Gentamycin (5 mg/kg IM) once a day was administered for 4 days after surgery. In order to prevent dehydration, a bolus of subcutaneous saline lactate was given, 20ml prior to surgery, 20ml at the completion of surgery, and 10ml twice a day for 3 post-operative days.

### CPP

Beginning at 20-week age (i.e., 3 weeks after the surgery), animals were conditioned in a three-chamber CPP apparatus [as previously described [28,29]; S1 Materials]. Thirty-minute conditioning sessions occurred during eight consecutive days, in which animals were separately conditioned to both bacon and chow (4 days in each, alternating every other day). A measured amount of the food stimulus (either cooked bacon or Purina chow, 5 g each) was placed in the stimuli’s paired-chamber 20 minutes into each conditioning session. The animal had free access to the food during the session’s remaining ten minutes, and after the experiment, bacon or chow consumption was measured. Beginning on day five, and in order to acclimate animals to the 2-fluoro-2-deoxy-_D_-glucose (FDG) during PET scans (details below), animals were injected with saline immediately prior to each conditioning session. Test days 1 and 2 lasted 15 minutes, and during these sessions, animals had unrestricted access to the two chambers and no food stimulus was provided (cues only).

### Small Animal Positron Emission Tomography (μPET)

**Fig 1:**
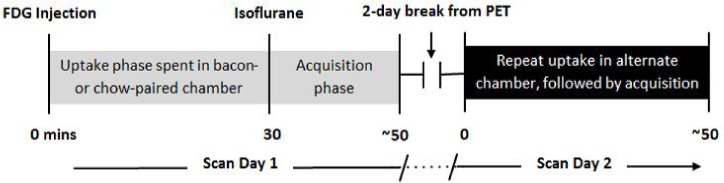
Scan day flow chart. Each animal was scanned twice, separately in the presence of either bacon- or chow-paired cues, but without either food stimulus. Animals were conscious during the uptake phase, and immediately prior to and during acquisition (20 min scan), animals were anesthetized with continuous isoflurane. During the 2-day break, CPP was maintained with one conditioning session per day (not shown).

Animals were scanned twice using in-vivo μPET, with FDG, to measure brain metabolism [17] [30]. Scans were separated by two days of continued CPP conditioning (conditioning days 11–12). On each scan day, animals were injected with 1 mCi FDG (i.p.) 30 minutes prior to the start of the acquisition (uptake period) (Fig 1). During the 30-minute uptake phase, the animals remained awake and in either the bacon- or chow-paired chamber (Fig 1). Then, animals were anesthetized with 1% isoflurane and scanned (20 minutes) using a ramp filter with cutoff at Nyquist frequency. During this time, non-fasting blood-glucose levels were measured from tail-vein blood using a glucometer (Truetrack, CVS).

### Procedure

During the FDG uptake period, rats were placed in either the bacon- or chow-paired chamber immediately after the injection (similar to the last 3 conditioning days where rats received saline injections). The placement was randomized so that half of the rats from each group were placed first in the bacon-paired chamber and then in the chow-paired chamber and the other half vice-versa. This paradigm was designed to mimic the conditioning session of the CPP procedure with the exception that chow or bacon was not administered.

### Image Reconstruction and Analysis

Images were spatially normalized and co-registered to a magnetic resonance image (MRI) atlas [17] using the PMOD software environment (PMOD Technologies, Zurich, Switzerland) and a custom in-house designed region of interest (ROI) template (regions identified using the Paxinos atlas) was then applied to the MRI atlas. Statistical Parametric Mapping (SPM) was used to examine changes in BGluM in regions not selected a priori with a few modifications to previously described methods [[31,32] S1 Materials].

## Results

**Fig 2:**
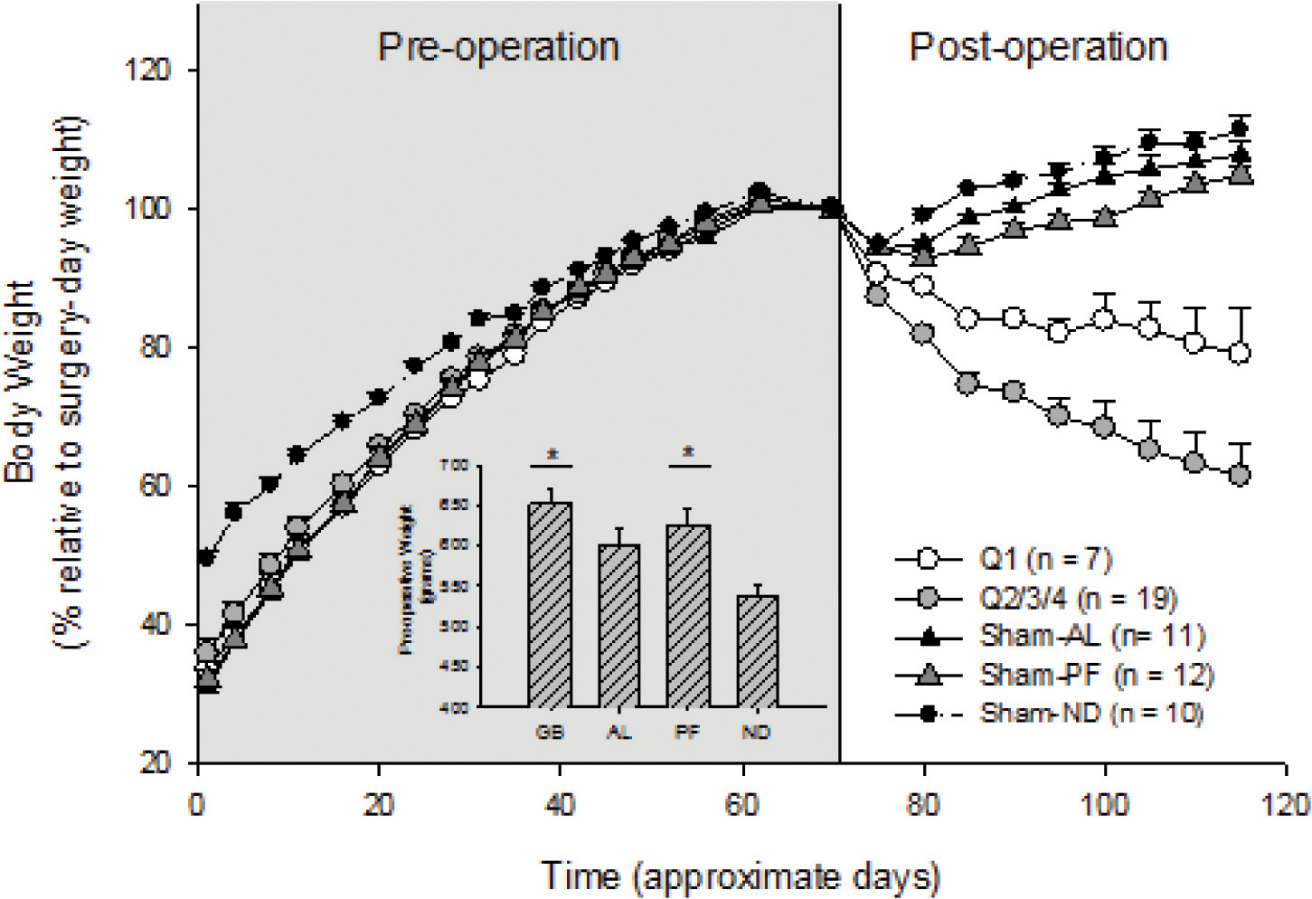
Gastric bypass effectively reduced body weight following diet-induced obesity. Pre-operative body weights during diet-induced obesity (establishment phase) are shown in the shaded region. Weight loss following surgery, specific to the RYGB animals, is shown in the non-shaded region, with a more pronounced difference in the Q2/3/4 sub-group. The inset shows the differences in body weights, following group-assignment, the day before experimental or sham surgeries (*p< 0.05 compared to Sham-ND; no difference between Q1 and Q2/3/4 before surgery, not shown).

Differences between HF- and ND-animals were noted five weeks after diet initiation; HF animals were significantly heavier at the time of surgery (Kruskal-Wallis Test: H_3_ = 16.78, *p*< 0.001; Fig 2, inset). Post-hoc comparisons (Dunn’s method) revealed that RYGB and Sham-PF rats were significantly heavier than Sham-ND (RYGB: Q = 4.03; Sham-PF: Q = 2.994; *p* <0.05). There were no differences amongst the three HF groups. After surgery, immediate weight decreases were observed in all groups, with the greatest decreases observed in the RYGB group (Fig 2). By the start of the behavioral and neuroimaging experiments, weight loss was only observed in the RYGB group, while in the sham-operated animals weight gain was observed. Rats with surgical complications were excluded based on one of the following parameters: altered appearance, grooming, bowel quality and movements. Necropsy results were assessed by the surgery team and veterinary technicians caring for the rats and were used to make a determination regarding the presence of surgical complications and decision to exclude was based on these independent assessments.

**Fig 3:**
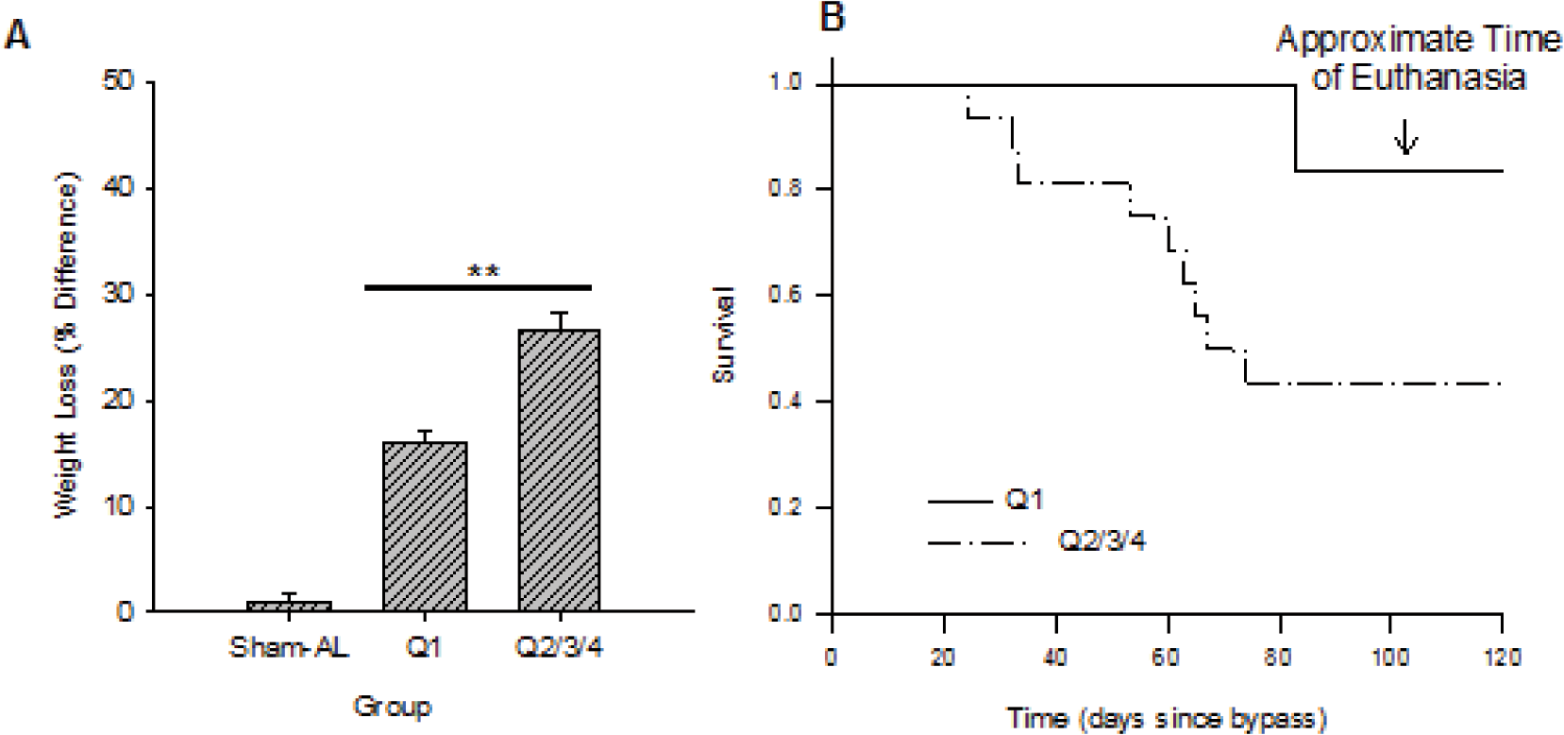
Objective health-screen and bypass outcome. As gross health and weight were highly variable in the experimental group, compared to the sham-operated controls, relative weight-loss after gastric bypass was calculated and used to objectively determine these animals’ health. **(A)** The 7 RYGB animals with the lowest and most stable weight-losses (Q1) were compared to the remaining 75% (**p< 0.01 compared to each other; Sham-AL shown as reference). **(B)** This objective separation was valid, as differences in mortality were explained by the screen.

The health of the RYGB animals was variable (appearance, grooming, diarrhea), as were their weights and survivals. To control for the effects of RYGB on health and weight loss, animals were objectively stratified into sub-groups that were analyzed separately. To accomplish stratification, percent weight loss was calculated relative to each subject’s pre-operative weight. These values, which assessed the stability of each subject’s body weight, varied within the RYGB animals (range: 11.3–48.9%) greatly exceeding the Sham-AL animals (Mann-Whitney Test: U = 49.00, *p*< 0.001, *n* = 11 and 32). The RYGB animals with the greatest weight-loss stability (25^th^ percentile, Q1, n = 7) likely responded best to the surgery, compared to the remaining 75% of this group (Q2/3/4, n = 19). In fact, weight-loss differed significantly between these two sub-groups (Mann-Whitney Test: U = 0.00, *p*< 0.001, *n* = 7 and 19; Fig 3A), yet before the operations, there was no difference in body-weight between them (Mann-Whitney Test: U = 37, *p* = 0.094, *n* = 7 and 19).

### Body Weight and Food Intake

Intra-group body weight was stable the week before and during CPP (time main effect: F_2,146_ = 2.954, *p* = 0.057), yet across the five groups, body weights differed significantly (group main effect: F_4, 146_ = 9.276, *p*<0.001; time × group interaction: F_8, 146_ = 4.642, *p*<0.001). Based on pair-wise comparisons (Holm-Sidak method), the body weight of the Q2/3/4 group was significantly less than for Sham-PF (*t* = 5.495, unadjusted *p*<0.001),-AL (*t* = 5.152, unadjusted *p*< 0.001),-ND (*t* = 3.326, unadjusted *p* = 0.002) groups and the Q1 group (*t* = 3.212, unadjusted *p* = 0.002).

**Fig 4:**
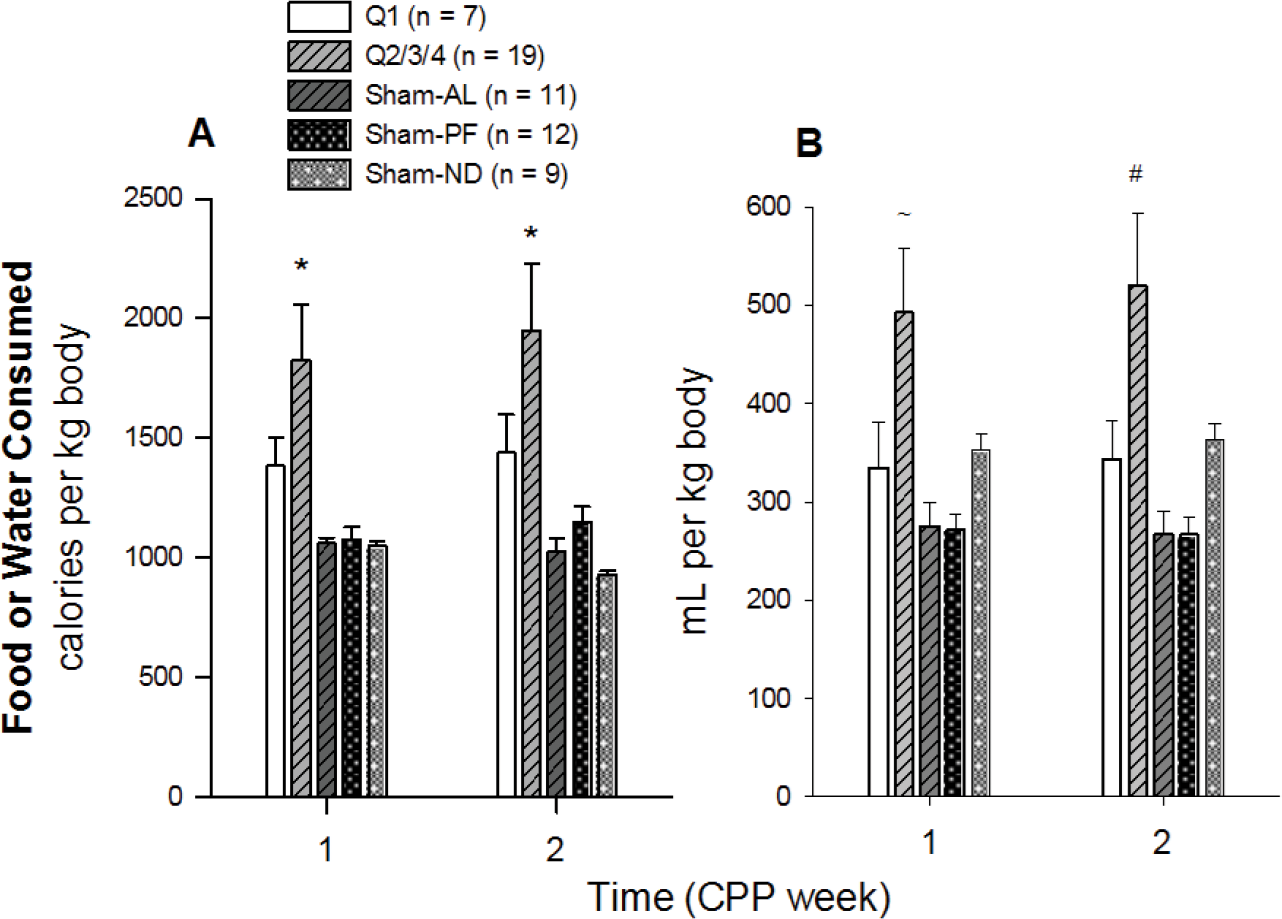
Weekly food and water intake during bacon- and chow-conditioning. Weekly food **(A)** and water **(B)** intakes were calculated relative to CPP Weeks 1 and 2, and these values, expressed in calories and volume consumed, respectively, were adjusted based on body-weight (*unadjusted *p* < 0.001 versus Sham-AL,-PF and-ND; ^~^unadjusted *p* < 0.001 versus Sham-PF and-AL; ^#^unadjusted *p* < 0.007 versus all groups).

Food intake was temporally stable during the two CPP weeks (time main effect: F_1, 96_ = 0.266, *p* = 0.609; time × group interaction: F_4, 96_ = 1.944, *p* = 0.120; Fig 4A), but it differed amongst the five groups (group main effect: F_4, 96_ = 8.762, *p*<0.001). Pair-wise comparisons (Holm-Sidak method) revealed that Q2/3/4’s intake was greater than that of Sham-AL (*t* = 4.885, unadjusted *p*<0.001),-PF (*t* = 4.435, unadjusted *p*< 0.001) and-ND groups (t = 4.936, unadjusted *p*<0.001). Intake did not differ from that of Q1’s (*t* = 2.444, unadjusted *p*<0.0019). As a consequence, Sham-PF did not differ from Sham–AL.

Intra-group water intake was also temporally stable during the CPP experiment (time main effect: F_1, 97_ = 0.814, *p* = 0.372; time ×group interaction: F_4, 97_ = 0.763, *p* = 0.555; Fig 4B) but differed amongst the five groups (group main effect: F_4, 97_ = 7.179, *p*< 0.001). Holm-Sidak post-hoc tests revealed that Q2/3/4’s water consumption was significantly greater than Q1 (*t* = 2.909, unadjusted *p* = 0.006), Sham-AL (*t* = 4.607, unadjusted *p*<0.001) and-PF (*t* = 4.753, unadjusted *p*< 0.001) groups, while all other comparisons were not significant (unadjusted *p* values >0.007).

### CPP

**Fig 5:**
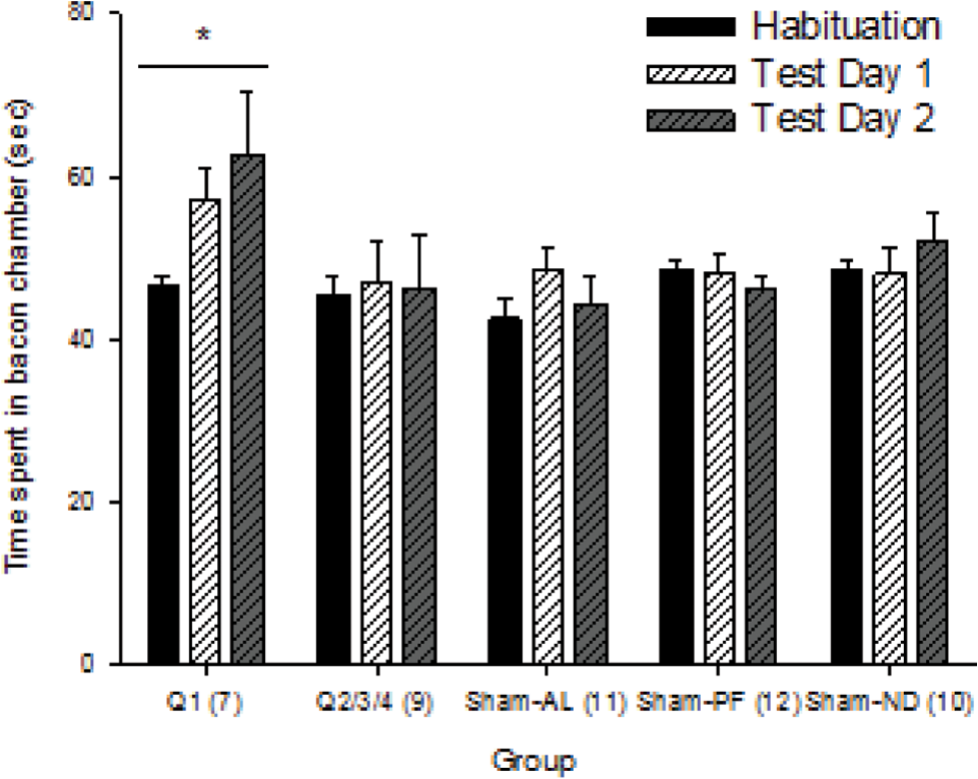
Healthy RYGB rats (Q1) showed significant bacon CPP. To quantify preference for the bacon-paired chamber, the amount of time each animal spent in the bacon-paired chamber was standardized to total time in both chambers. There was a bypass-specific increase in bacon-chamber preference after conditioning (Test Days 1 and 2) relative to Habituation Day, yet importantly, this behavioral response was strictly observed in the RYGB animals that responded well to the surgery (*unadjusted *p* < 0.05 compared to Habituation Day).

**Fig 6:**
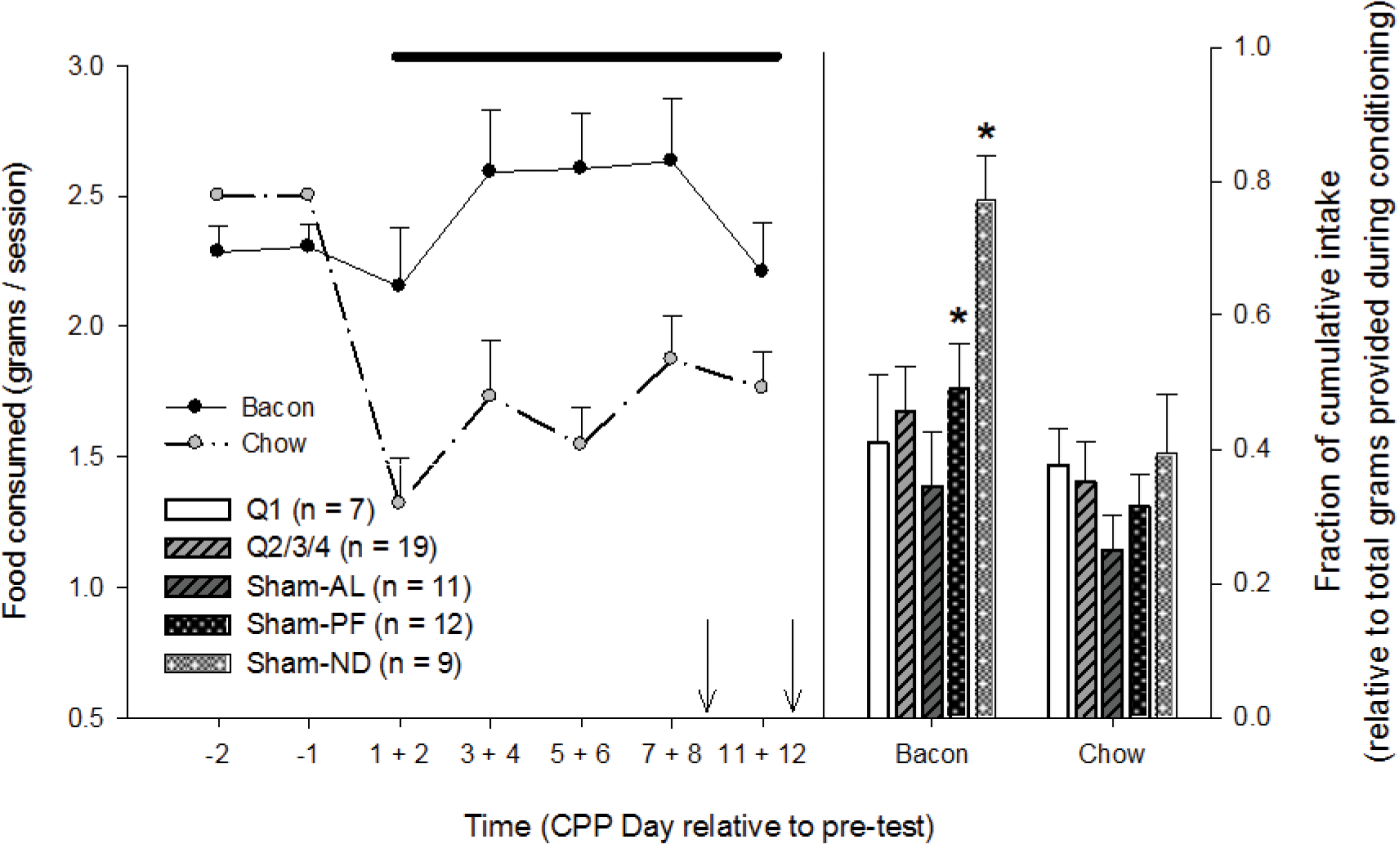
Food intake throughout the conditioning sessions. Animals were given 10-minutes to consume 5-grams of either bacon or chow during the final 10 min of each conditioning session. Irrespective of group, animals consumed more bacon than chow throughout the conditioning-component, suggesting that relative to chow, bacon was highly palatable. Cumulative intake is the sum grams consumed between CPP days 1 and 12 for each food-stimulus (arrows indicate Test Days 1 and 2; thick line: *p* < 0.05 for bacon versus chow on the given days; *p < 0.05 for bacon versus chow in a specific group). The only significant differences observed were found between bacon and chow (Sham-ND and Sham-PF).

To determine bacon-cue preference (time spent in the bacon chamber, relative to total time exploring), we compared CPP between test-sessions and across groups. Bacon-cue preference was significantly different between pre- and post-conditioning trials (time main effect: F_2, 157_ = 4.575, p = 0.013; Fig 5). There were no differences in CPP between groups (group main effect: F_4, 157_ = 1.981, p = 0.112; time × group interaction: F_8, 157_ = 1.538, p = 0.155). Post-hoc comparisons (Holm-Sidak method) revealed differences only in Q1 group, who showed significantly greater CPP after conditioning (Fig 6), when compared to Habituation Day (Test Day 1: t = 3.517, unadjusted p <0.001 and Test Day 2: t = 2.467, unadjusted p = 0.015; n = 7).

### Bacon Consumption

There was a trend for increased bacon consumption throughout conditioning relative to pre-conditioning (Days -2 and -1), which was driven by the Sham-ND group. For cumulative food intake throughout conditioning, there were significant effects for food (main effect: F_1,97_ = 31.316, p<0.001), group (main effect: F_4,97_ = 2.814, p = 0.037) and the interaction (food × group interaction: F_4,97_ = 4.136, p = 0.006); Fig 6. Post-hoc comparisons (Holm-Sidak method), showed increased bacon intake, compared to chow intake, in the Sham-PF (t = 3.122, unadjusted p = 0.003) and Sham-ND (t = 5.867, unadjusted p<0.001) animals. There were no differences in bacon vs. chow intake in the RYGB rats.

### μPET (SPM Analysis)

**Fig 7:**
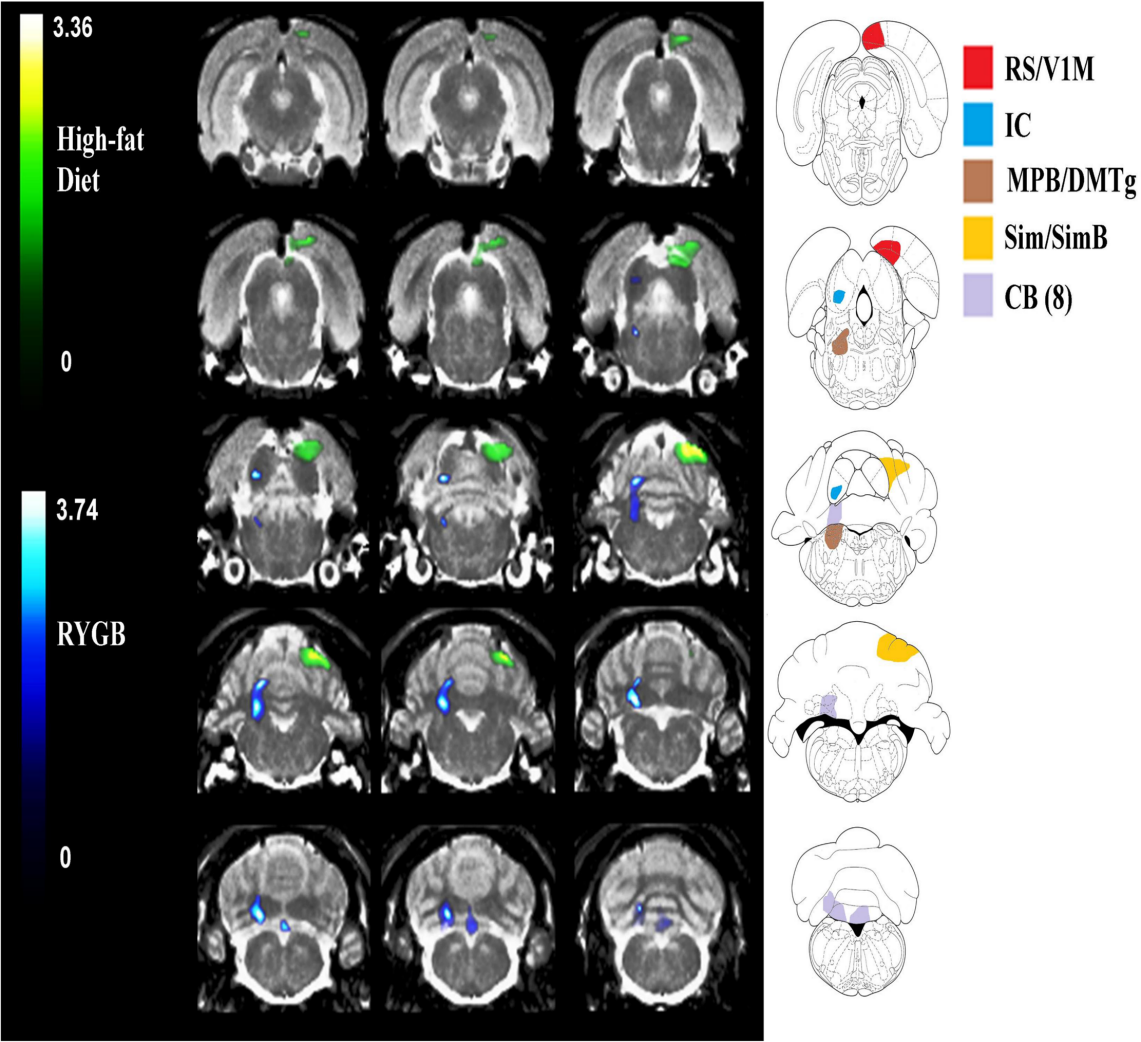
Statistical Parametric Mapping (SPM) showing regional brain activation to bacon-paired cues in rats that underwent gastric bypass surgery (RYGB; blue) or sham-operated controls fed a HF diet (green). SPM clusters were overlaid onto a magnetic resonance imaging atlas of the rat brain set to stereotaxic coordinates and significant clusters were outlined in the Paxinos rat brain atlas. (RS-retrosplenial cortex; V1M-primary visual cortex; IC-inferior colliculus; MPB-medial parabrachial nucleus; DMTg-dorsomedial tegmental area; Sim/SimB- simple lobule; CB(8)-cerebellum lobule 8).

**Fig 8:**
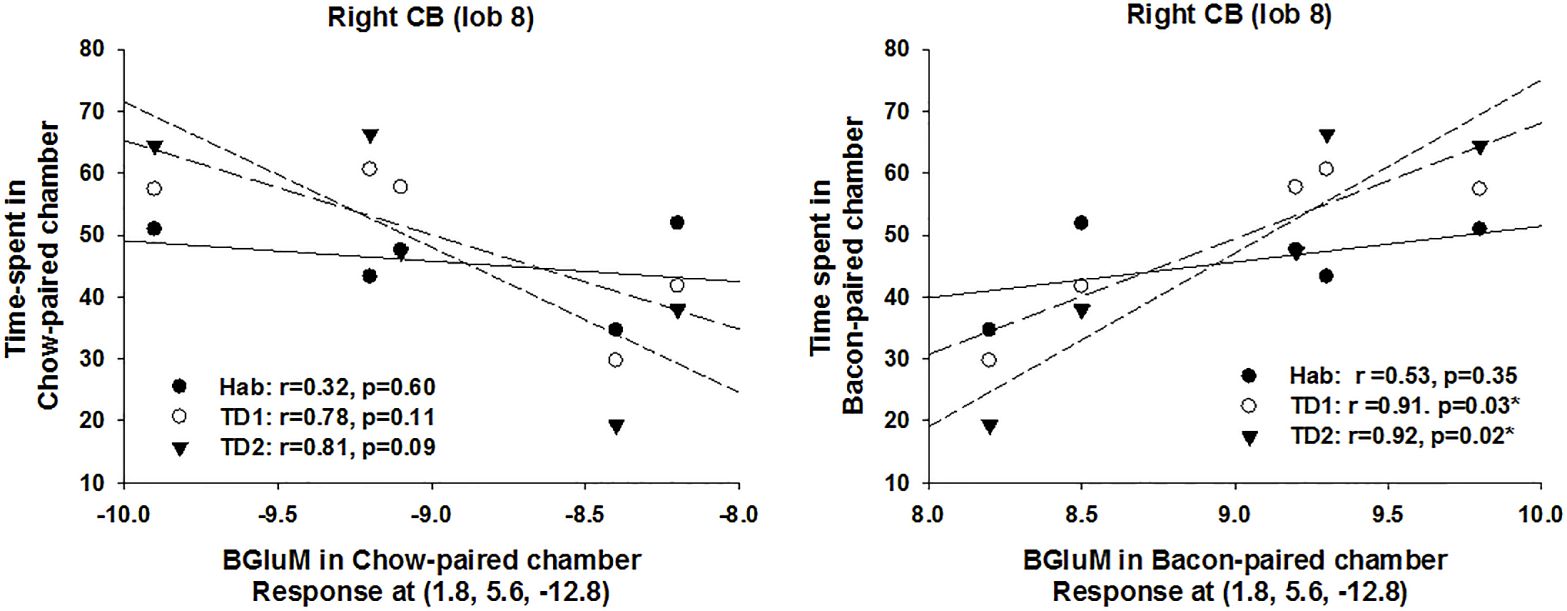
Correlation plots between CPP preference for Chow or Bacon and BGluM during Habituation (Hab), Test Day 1 (TD1) or TD2.

Due to technical limitations (motion artifacts during scanning), 7 animals were excluded (1 from Q1, 1 from Q2/3/4, 3 from Sham-PF and 2 from Sham-AL). SPM analysis revealed significant differences for the contrast bacon > chow for RYGB and AL (high-fat) groups (Fig 7 and Table 1) but no differences for the contrast chow > bacon for any of the groups. For RYGB rats, one cluster was observed that included the right hindbrain (pons), the right and midline cerebellum and the medial parabrachial (MPD) and dorsomedial tegmental area (DMTg). For AL rats, one cluster was observed that included the left CB (Sim), CB (SimB), and retrosplenial area (RS) and the right primary visual cortices (V1M). For RYGB rats, regression analysis showed that BGluM in right cerebellum to bacon-paired cues predicted preference for these cues. That is, individual responses in the right CB (lob 8) during exposure to the bacon-paired chamber were found to significantly correlate with preference for this chamber on both Test Days 1 and 2 but not during Habituation (Fig 8). No other significant correlations were observed.

**Table 1:** SPM Results.

**RYGB (Chow < Bacon)**							
**cluster-level**			**peak-level**				
**P_FWE-corr_**	**q_FDR-corr_**	**K_E_**	**P_uncorr_**	**T**	**Z**	**mm mm mm**	**Region**
0.01	0.01	1390	<0.001	41.32	4.75	1.8 5.6–12.8	Right Cerebellum (lob. 8)
			<0.001	19.33	4.1	2.0 7.4–8.6	Right MPB, DMTg
			<0.001	18.53	4.05	0.5 5.5–13.0	Midline Cerebellum (lob. 8)
**High-Fat Diet (**AL) **(Chow < Bacon)**							
**cluster-level**			**peak-level**				
**P_FWE-corr_**	**q_FDR-corr_**	**K_E_**	**P_uncorr_**	**T**	**Z**	**mm mm mm**	**Region**
0.022	0.015	1499	<0.001	16.02	4.3	2.2 1.4–7.4	Retrosplenial cortex (RS)/Left Primary visual cortex (V1M)
			<0.001	15.9	4.29	3.4 2.5–9.8	Left Cerebellum (Sim)
			<0.001	14.8	4.21	4.0 3.5–10.2	Left Cerebellum (SimB)

Statistical Parametric Mapping (SPM) results showing significant clusters and statistical parameters for the contrast Bacon>Chow in rats that underwent gastric bypass surgery (RYGB) and sham-operated controls (AL). The contrast Chow>Bacon did not yield any significant clusters. (MPB) medial parabrachial nucleus, (DMTg) dorsomedial tegmental area (Sim) simple lobule.

## Discussion

As expected, RYGB animals lost weight, while sham-operated HF-fed animals continued to gain weight [33,34]. However, we found that RYGB rats consumed more chow than the other groups, opposite from the findings of other authors, reporting food intake decreases after RYGB [34,35]. Since the RYGB reduces the stomach size, and in turn meal size, an overall increased food intake may be explained with increased meal frequency (small amounts but more frequently throughout the day) to compensate for malabsorption, another characteristic of RYGB [25]. Alternatively, theoretically there could have been dilation of the gastric pouch and Roux limb, which in conjunction with the eating habits might account for the observed increase in food consumption in the RYGB group (38). For example, in rats, the size of the gastric pouch and luminal diameter of the post-anastomosis jejunum increases in size as early as 11 days post-operatively [35]. Notably, human RYGB patients also increase food intake in the months following their surgery [36]. Finally, we did not specifically measure activity patterns in this experiment. Certainly this is a parameter, which affects weight loss in actual patients and contributes to success and/or failure of weight loss. This limitation will need to be addressed by future studies.

Only the healthy (Q1) RYGB group showed increased preference for the bacon-paired chamber (Fig 5). This suggests that the RYGB procedure may have enhanced the conditioning properties of bacon even when it was not accompanied by an increase in its consumption. In contrast, the Sham-PF and ND animals did not demonstrate bacon CPP yet they increased their bacon intake throughout the conditioning process. To explain these differences, the RYGB (Q1) rats may have been restricted from increasing their bacon intake due to the RYGB procedure since the bacon was only present for a short time period (fifteen minutes) and their feeding is characterized by small sampling over prolonged time periods.

Previous sham-controlled studies reported that RYGB decreased preference for high concentrations of sucrose but increased preference for lower concentrations [33,37]. Literature on RYGB in humans and rodents demonstrates a shift in preference towards fatty and sweet foods of lower concentrations in contrast to obese individuals who prefer higher sweetness concentrations [24,37]. Previous studies have not been consistent with respect to the RYGB effects on fat consumption with one study showing no changes [38], whereas recent studies showed decreases in the preference for high fat food [25,37,39]. Furthermore, the magnitude and durability of the reported RYGB-related changes in food selection show significant inconsistencies [40], and pre-surgical exposure to fat may also influence post-operative food preferences [41].

RYGB may affect both taste preferences and actual preferential intake of different macronutrients, and it is important to distinguish between an increase in liking or disliking a taste despite the absence in increases in intake, due to the restrictive nature of the RYGB [42]. In obese rats, RYGB alleviates obesity-related alterations in taste function, namely shifting preference from high to lower fat concentrations. RYGB animals also change their responses to fatty food; and show a preference for a diet with low rather than with high-fat content [25]. Moreover the sensitivity to the rewarding properties of HF foods was reduced after RYGB [24,25]. Furthermore, RYGB in rats showed altered gene expression involved in regulating food intake in the hypothalamus [43].

Prior studies reported that animals fed only normal chow ignore high-fat diets [44]. We show that following habituation to an extremely high-fat diet (60% kcal from fat), the RYGB increased conditioning to a fatty food such as bacon, which was not observed in the other animals. However, the RYGB rats despite their increased conditioned responsiveness did not increase their consumption of bacon, whereas the rats in the other groups increased their consumption of bacon even when they did not show increased CPP. This dissociation between CPP and consumption highlights the fact that these two behaviors do not necessarily reflect the same neurobiological processes. A similar dissociation between striatal brain activity related to impulsivity to seek and consume food reward has been found in overweight individuals [18]. Conditioning reflects associative learning linked with a rewarding stimulus whereas consumption reflects the motivational drive to perform the behavior to get the reward. Indeed, animals can be divided into those that respond to cues (sign-trackers) versus those that respond to the reward (reward-trackers)[45]. Thus, it is possible that RYGB enhances anticipatory food reward, and in turn, sign tracking behavior to food-cues.

### Increased Cue-Reactivity and Its Potential Implication for Increased Risks for Weight Regain

In this study, the RYGB rats with the least weight-loss (25th percentile, Q1) responded to the surgery with the least weight loss compared to the remaining 75% of rats in this group. Following RYGB, whereas most patients lose a substantial amount of (60–70%) excess body weight and voluntarily restrict consumption of calorie-dense, highly palatable foods, and report fewer cravings [39,46–49]; about one fifth of the patients [50] lose less weight and/or regain their initial weight loss within five years after surgery. It is hypothesized that the RYGB patients with a prior history of binge eating may be more susceptible to poor surgical outcome [51]. Binge Eating Disorder (BED) is recognized as a diagnosis in the new DSM-5 [52], and afflicts 1 in 35 American adults [53]. Approximately 35% of those who regularly binge-eat are overweight or obese, and are at a higher risk for hypertension, dyslipidemia and type-2 diabetes [54]. Therefore, it is not surprising that 10–27% of RYGB surgery patients meet criteria for BED[53,55–57]. It has been proposed that occurrence of certain BED symptoms (e.g., food cravings, loss of control over eating, nibbling or nighttime eating) may indicate a risk for weight regain in the years after bariatric surgery [51,58–62]. Imaging studies suggests that following RYGB, food-cues may elicit reduced activation in brain areas [63]. RYGB appears to result in blunted activation in the dorsolateral prefrontal cortex [63,64] that is considered to be an area involved in inhibiting impulsive behaviors. These and other data collectively suggest a greater risk for some patients to engage in alternative excessive behavior [65]. Clinical observations suggest an increased risk among RYGB patients for substituting food with alcohol [66–69] or use of other substances [70,71] and a history of alcohol abuse represents a relative contraindication for most bariatric surgery programs [72]. Recently, we [73–75] and others [76] reported increased alcohol self-administration in RYGB rats to either oral or intravenous routes of administration. As an additional concern, salient parallels exist in the clinical presentations of BED and food addiction [77] as well as substance dependence [78,79]. Recent studies have found that bariatric surgery candidates with BED displayed addictive personalities [80,81]. As addictive personality scores increase, overeating behaviors also increase. In this context, one alternative interpretation of the current findings is that the subgroup of the RYGB rats that lost the least body weight (Q1) showed the greatest behavioral sensitivity to form conditioned preference for food-cues. Furthermore, the same cohorts showed the highest brain activation to the same food-cue compared to those animals that lost more weight and maintained it over time. Should this speculation be correct, one may expect that RYGB patients with lower loss of weight or weight regain during the first few years would also display increased cue-reactivity when tested in behavioral tasks or scanned with functional brain imaging. Future studies are warranted to confirm such a relationship.

### Enhanced Brain Activation in Response to Bacon

Bacon anticipation activated distinct parts of the cerebellum in both RYGB and AL rats. The cerebellum is involved with reward-based reversal learning [82] expected utility [83] and predictive value and reward processing [84]. As brain activity was assessed in response to palatable food anticipation, and individual subject activation in cerebellum significantly predicted bacon preference. These findings suggest that RYGB selectively affects specific parts of the cerebellum (right and midline cerebellum; Lob 8) involved in subjective processes related to reward or value expectation.

The cerebellum receives dopaminergic, serotonergic, noradrenergic, and cholinergic inputs that perform functional modulation of the brain [85]. Sensorimotor tasks activate anterior cerebellum (lobule 5, 6 and 8); while motor activation is observed in lobules 8 [86]. The relationship between obesity and cerebellar structural and functional deficits has been reported and may be mediated by several potential mechanisms [52,87–89], including increased cerebellar microglial activation [90] and cerebellum neuronal injury [increased gene expression of neuron specific enolase; [87]]. Increased leptin is associated with disrupted cerebellar activity [52] and the cerebellum plays an important role in regulating leptin-mediated processes related to food intake. The cerebellum shows strong leptin receptor gene expression [91] and is activated by circulating leptin and diet-induced obesity, which forms a monitoring/signaling pathway to complement more direct hypothalamic interactions [92].

In addition, bacon anticipation in RYGB rats also activated an area approximating the medial parabrachial nuclei (MPB) and the dorsomedial tegmental area (DMTg). The MPB is involved in sensory representation related to gustatory processing [93,94] whereas the DMTg has been implicated in reward processing[95]. Therefore, our findings suggest that abnormal brain activity in these areas may underlie changes in taste and reward related processes after RYGB [26,33,39,40,96–99]. Interestingly, bacon anticipation activated a very different set of regions in AL rats from those observed in RYGB rats. The RS and V1M activation in response to bacon anticipation in AL rats may be attributed to memory-related aspects [100,101] associated with the conditioned environments for bacon and chow anticipation, which were contextually very different.

These findings may hold important clinical implications. In fact, these results, together with recent reports of increased susceptibility to non-food rewards, such as increased use of alcohol [27,75] suggest an increased risk for some patients to choose a replacement reward after RYGB. As such, our study provides a possible preliminary neurobiological explanation of why patients may increase the consumption of alternative rewarding substances after RYGB [66]. It also needs to be pointed out that the possibility exists that most highly obese individuals get that way because of Reward Deficiency Syndrome (RDS) having neurogenetic and epigenetic effects in terms of the entire brain reward system including prefrontal cortex executive function dysregulation due to hypodopaminergic trait/ states (103).

In summary, RYGB alters reactivity of areas involved in reward expectation and sensory processing when subjects anticipate a fatty highly palatable food such as bacon. Moreover cerebellum response to food cues in the RYGB rats predicted their CPP responses; identifying the cerebellum as a region that may mediate some of the changes in eating behaviors that follow RYGB. Overall, these observations suggest that despite reduced food intake and improved food choices after RYGB, brain areas that are involved in reward anticipatory functions may be more active.

## Supporting Information

S1 Materials Supplemental information and references regarding methodology. (DOCX)
