# Supplementary material for: Roux-en-Y Gastric Bypass Alters Brain Activity in Regions that Underlie Reward and Taste Perception
Source: PLoS One. 2015 Jun 3;10(6):e0125570. doi: 10.1371/journal.pone.0125570 (PMC4454506; doi:10.1371/journal.pone.0125570)
Supplement: S1 Materials — (DOCX) [file pone.0125570.s001.docx]

# S1 Materials

**Animals:**

All experiments were conducted in conformity with the National Academy of Sciences Guide for Care and Use of Laboratory Animals [[1](#_ENREF_1)]. All work was approved by the Penn State University College of Medicine and Brookhaven National Laboratory Institutional Animal Care and Use Committees.

**CPP:**

The central corridor (12 cm × 20 cm × 20 cm) linked two equally sized chambers (30.5 cm × 20 cm × 20 cm), and these chambers were distinguished on the basis of wall color, pattern and flooring. All trials were conducted between 1400 and 1800 hrs. For pre-conditioning, animals were placed in the middle corridor, and then given unrestricted access to both chambers for 15mins. If an animal showed obvious preference for one chamber over the other (≥55% time spent), it received bacon in the opposite, non-preferred chamber during the conditioning phase. When an animal was unbiased (<55% time spent), that rodent’s bacon-paired chamber was randomly assigned.

**Small Animal Positron Emission Tomography (µPET**).

We used a μPET R4 tomograph (Concorde CTI Siemens, Knoxville, TN; transaxial resolution of 2.0mm full-width at half maximum, with a field-of-view of 11.5 cm).

**Image Reconstruction and Analysis:**

Images were reconstructed using the maximum-a-posteriori (MAP) algorithm (20 iterations, 0.01 smoothing value, 256x256 resolution) with reconstruction voxel sizes of x=0.42 x y=0.42 x z=1.21 mm. The ROI template consisted of the following ROIs: olfactory bulb, frontal cortex, cingulate cortex, orbital cortex (OR), insular cortex, parietal cortex, nucleus accumbens (Nacc), striatum (ST), occipital cortex, temporal cortex, hippocampus, thalamus (TH), hypothalamus (HYP), primary somatosensory cortex, and cerebellum. FDG uptake values were reported in kBq/cc and normalized for injected dose, body weight and blood glucose levels as described [[2](#_ENREF_2)].

Statistical Parametric Mapping (SPM) was performed on images which were co-registered and spatially normalized in the Fusion module of PMOD to an MRI template of the rat brain set to Paxinos and Watson stereotaxic coordinates [[3](#_ENREF_3)]. This type of “anatomical” spatial normalization increases the spatial specificity since the greater resolution of the MRI improves normalization at a finer scale [[4](#_ENREF_4)]. After the registration and normalization process, images were smoothed (4 mm Gaussian) and analyzed using SPM8 and the results overlaid onto the stereotaxic MRI template using the Fusion module in PMOD. All contrasts were evaluated at the p<0.05 level after correcting for multiple comparison using false discovery rate (FDR) and family-wise error parameters (FWE).

**References**

1. NAS, NRC (1996) Guide for the Care and Use of Laboratory Animals. Washington D.C.: National Academy Press.

2. Thanos PK, Michaelides M, Piyis YK, Wang G-J, Volkow ND (2008) Food restriction markedly increases dopamine D2 receptor (D2R) in a rat model of obesity as assessed with in-vivo μPET imaging ([11C] raclopride) and in-vitro ([3H] spiperone) autoradiography. Synapse 62: 50-61.

3. Schweinhardt P, Fransson P, Olson L, Spenger C, Andersson JL (2003) A template for spatial normalisation of MR images of the rat brain. J Neurosci Methods 129: 105-113.

4. Gispert JD, Pascau J, Reig S, Martínez-Lázaro R, Molina V, et al. (2003) Influence of the normalization template on the outcome of statistical parametric mapping of PET scans. NeuroImage 19: 601-612.
